# Brief relaxation training is not sufficient to alter tolerance to experimental pain in novices

**DOI:** 10.1371/journal.pone.0177228

**Published:** 2017-05-11

**Authors:** Karen E. Smith, Greg J. Norman

**Affiliations:** 1 Department of Psychology, Integrative Neuroscience Area, University of Chicago, Chicago, Illinois, United States of America; 2 Grossman Institute for Neuroscience, University of Chicago, Chicago, Illinois, United States of America; National Natural Science Foundation of China, CHINA

## Abstract

Relaxation techniques, such as deep breathing and muscle relaxation, are aspects common to most forms of mindfulness training. There is now an abundance of research demonstrating that mindfulness training has beneficial effects across a wide range of clinical conditions, making it an important tool for clinical intervention. One area of extensive research is on the beneficial effects of mindfulness on experiences of pain. However, the mechanisms of these effects are still not well understood. One hypothesis is that the relaxation components of mindfulness training, through alterations in breathing and muscle tension, leads to changes in parasympathetic and sympathetic nervous system functioning which influences pain circuits. The current study seeks to examine how two of the relaxation subcomponents of mindfulness training, deep breathing and muscle relaxation, influence experiences of pain in healthy individuals. Participants were randomized to either a 10 minute deep breathing, progressive muscle relaxation, or control condition after which they were exposed to a cold pain task. Throughout the experiment, measures of parasympathetic and sympathetic nervous system activity were collected to assess how deep breathing and progressive muscle relaxation alter physiological responses, and if these changes moderate any effects of these interventions on responses to pain. There were no differences in participants’ pain tolerances or self-reported pain ratings during the cold pain task or in participants’ physiological responses to the task. Additionally, individual differences in physiological functioning were not related to differences in pain tolerance or pain ratings. Overall this study suggests that the mechanisms through which mindfulness exerts its effects on pain are more complex than merely through physiological changes brought about by altering breathing or muscle tension. This indicates a need for more research examining the specific subcomponents of mindfulness, and how these subcomponents might be acting, to better understand their utility as a clinical treatment.

## Introduction

Relaxation interventions, such as deep breathing or muscle relaxation, have demonstrated efficacy in treating pain symptoms in patients suffering from chronic pain [1], and there is some evidence that they are also effective in treating clinical disorders, including anxiety [2] and depression [3]. These techniques are similar to what are termed mindfulness techniques. While there are a range of definitions, the key distinction made between relaxation and mindfulness is that there is an intentional focus to relax during the practice of relaxation methods while mindfulness involves the cultivation of a non-judgmental, moment-to-moment awareness [3]. However, it is not clear how different the two types of interventions are in terms of efficacy. Studies which have directly compared them have found relatively similar effects on anxiety and mood [3,4]. Indeed, there has been a trend towards interventions which incorporate both mindfulness and relaxation techniques in combination, often broadly referred to as “mindfulness based” interventions [5]. These interventions have demonstrated efficacy for treating a wide range of disorders, including many debilitating clinical conditions, such as depression, anxiety, borderline personality disorder, eating disorders, and chronic pain [5–8], leading to their increasing use within clinical populations. Given the often substantial efficacy of mindfulness interventions across a wide range of clinical conditions, many of which historically have been resistant to treatment, they offer an exciting new treatment tool within the clinical community.

One area in which there has been substantial research on the efficacy of mindfulness interventions is in individuals suffering from chronic pain [9,10]. Numerous anecdotal reports and case studies on long-term meditation practitioners have reported a significant reduction in self-reported pain symptoms [11,12]. Because of this, there has been a wealth of research examining the utility of mindfulness interventions for alleviating chronic pain symptoms [9,13–15]. As with other clinical areas, this work suggests there are positive effects of mindfulness interventions for chronic pain patients. However, this evidence is mixed, and indeed a recent systematic literature review found that there were no added benefits of mindfulness based pain interventions compared to cognitive and behavioral therapy alone [13]. Additionally, there is a range in what aspects of pain experiences are positively influenced by mindfulness based interventions, with the largest effects often seen for depression or negative mood associated with pain, while effects for self-reported pain are more mixed, with studies often finding no change in measures asking generally about participants’ current level of pain (i.e. How much pain are you in at the moment?) [16]. This suggests that these interventions have varying effects on different aspects of pain, and indicates a need for elaboration of what these aspects are and the mechanisms through which they are influenced. In order to better understand the mechanisms through which mindfulness produces symptom relief in chronic pain patients, it is important to examine how these types of activities affect acute experiences of pain in healthy participants, both at the level of nociception and self-reported experiences of pain, as well as any changes in general perceptions of stress or mood in response to the pain.

Compared to the literature on chronic pain, there are very few studies looking at the efficacy of mindfulness interventions on experiences of pain in healthy individuals [17,18]. Those studies that do look at the efficacy of mindfulness interventions on experiences of pain in healthy individuals have found mixed results [17,19–23]. Often these studies focus on current practitioners of mindfulness [19,22], making it difficult to compare how effective these interventions might be with novices or even how much training is necessary to see the observed effects. Additionally, it is possible that people who have higher tolerances for pain initially are more likely to practice mindfulness. However, there is some evidence that short one-time interventions may have beneficial effects in novices on nociception, or the response of the sensory nervous system to a noxious stimulus that leads to the subjective experience of pain [17,21,23]. This evidence is mixed though, with some research finding mindfulness increases both pain tolerance and self-reported pain [15,23], some finding changes in only the nociceptive response but not self-reported pain [21], and some finding no effects in novice participants at all [19].

The lack of consistent results for the effects of mindfulness interventions on experiences of pain is partially due to the variability across studies in the type of mindfulness or mindfulness subcomponent employed. Mindfulness is commonly defined as the quality of awareness that arises through intentionally attending to present moment experience in a non-judgmental and accepting manner [24]. However, the procedures used to achieve mindfulness in the literature include quite disparate practices ranging from deep breathing exercises [20,25] and muscle relaxation [26,27] to massage [28,29] and teaching full meditation traditions [30,31]. This variability in methodology across studies has made it difficult to determine which aspects of these interventions are associated with positive effects. Given this, it is important to investigate different components of mindfulness separately in order to better understand the mechanisms through which specific components may be producing positive effects. One approach to disentangling these mechanisms is to focus on the different subcomponents of these interventions and their specific mechanisms of action.

This study aims to provide insight into the effects of different mindfulness subcomponents, specifically focusing on two common relaxation components often employed within the context of mindfulness interventions: modulating breathing through a deep breathing task and gradual muscle relaxation through a progressive muscle relaxation task. These aspects were chosen because they are most common across the mindfulness intervention literature, with all practices involving some sort of instruction about slowing or attending to breathing, and many also incorporating a type of muscle relaxation task, often in the form of a body scan. Additionally, both of these tasks have been employed with and shown efficacy in novices [20,21]. However, these tasks have never been directly compared, and, while they appear to be effective at alleviating pain, the specific mechanisms through which this occurs are still unclear. These tasks are also easier to convey to individuals unfamiliar with the techniques as compared to more abstract aspects of mindfulness such as developing a state of nonjudgmental awareness of cognitions and streams of thoughts. Lastly, each of these tasks involves explicit instructions for modulating aspects of physiological function, which could influence both nociceptive and central experiences of pain. For example, changes in breathing modulate baroreceptor activity, stretch receptors which regulate blood pressure through alterations in sympathetic and parasympathetic cardiac control [32] and have been related to differences in pain sensitivity [33,34]. Additionally, muscle relaxation is thought to exert its effects through decreased afferent neural impulses from the skeletal musculature resulting in decreased sympathetic activity and reduced activity of neuromuscular circuits involved in the experience of pain [35,36]. These provide a concrete mechanism through which these types of interventions may influence individuals’ pain sensitivity.

The goal of this study was to assess how different relaxation aspects of mindfulness influence individuals’ experiences of acute pain and if they have differential effects. Additionally, this study aimed to examine the potential mechanisms through which the different relaxation aspects of mindfulness act. To do this, we compared the effects of a deep breathing, progressive muscle relaxation, and an active control condition on individuals’ pain tolerance as assessed by a cold pressor task. Throughout the study, we collected measures of cardiac parasympathetic and sympathetic nervous system activity to assess whether changes within these systems moderate any observed effects. We expect that both breathing and progressive muscle relaxation interventions will result in higher pain thresholds, We also expect that these increases will be related to physiological changes induced by the intervention. However, in contrast with deep breathing, we expect progressive muscle relaxation might produce a dissociation between pain tolerance and self-reported pain, with pain tolerance increasing without comparable changes in self-reported pain as previously observed [21].

## Materials and methods

### Participants

63 (24 male) University of Chicago undergraduates, ages 18–23 years (mean 20) participated in the study (22% Asian American, 11% African American, 0.02% Middle Eastern/Arab American, 41% Caucasian, 19% Multiple Ethnicities, 0.05% Other Ethnicity). Sample size was comparable to that in previous studies [20–23]. The participants were provided monetary or course credit compensation for their participation. Participants gave written informed consent, and this study was approved by the University of Chicago’s Institutional Review Board and conducted in accordance with the Declaration of Helsinki.

### Procedure

After arrival at the laboratory, participants were consented for the study and sensors were connected for all physiological measures. Participants then sat quietly for 5 minutes as an initial baseline assessment of physiological measures. After this baseline period, participants completed a set of questionnaires assessing demographics and current psychological state. Participants were then assigned to one of three 10 minute experimental conditions: deep breathing, progressive muscle relaxation, or a control condition. After the experimental condition, participants completed a short set of post-questionnaires. Participants then performed a cold pressor task.

### Questionnaire measures

Participants completed six questionnaires prior to undergoing the experimental condition, which included a demographic questionnaire; the State and Trait Anxiety Index (STAI) [37], a 20-item scale assessing individuals’ levels of state and trait anxiety; the Perceived Stress Scale (PSS) [38], a 10-item scale assessing individuals’ perception of stress, control, and predictability over life events in the past month; the Center for Epidemiological Studies—Depression Scale (CESD) [39], a 20-item scale assessing individuals’ feelings of depression; the UCLA Loneliness Scale [40], a 20-item scale assessing individuals’ perceptions of loneliness; and the Body Perceptions Questionnaire [41], a 122 item scale aimed at assessing individuals’ awareness of different body processes. Post experimental condition, participants again completed the PSS and the Trait portion of the STAI to assess any changes in perceived stress and anxiety after the different breathing conditions.

### Conditions

Participants were randomly assigned to one of three 10 minute experimental conditions: deep breathing, progressive muscle relaxation, or control condition. During the deep breathing condition, participants were instructed to match their breathing to a moving dot on the presentation—inhaling with the dot as it moved up, pausing as the dot remained flat, and exhaling as the dot moved down. This task was modeled upon previous deep breathing tasks [20,42,43] and was designed to reduce breathing to 5 breaths per minute. The progressive muscle relaxation consisted of a 10 minute audio segment, taken from one previously employed [21], during which participants were instructed to progressively tense and relax different muscle groups, while breathing deeply throughout. Lastly, in the control condition, participants were instructed to only watch the moving dot from the breathing condition without any explicit instruction to change breathing.

### Cold pressor

During the cold pressor, participants immersed their left foot in circulating cold water and ice slush maintained at 0°C. Participants were told to remove their foot when they could no longer tolerate the pain. If participants had not removed their foot by 5 minutes, the task ended, and the experimenter asked the participants to remove their foot. Previous work has utilized time cutoffs between 1–5 minutes [23,44–47]. We chose 5 minutes as a cutoff to ensure there was significant variability in participants’ pain tolerances—the length they were able to keep their foot in the water—as this was our primary outcome of interest. The amount of time (mm:ss) the participants kept their foot in the water was used as a measure of pain tolerance [48]. Additionally, participants were asked to rate the amount of pain they were experiencing every 30 seconds using a Visual Analogue Scale (from no pain to the worst imaginable pain), as has been employed previously [48]. The cold pressor task was only conducted once, post experimental condition, to avoid any potential habituation effects to the paradigm and to avoid inducing a state of stress in participants, via exposure to pain, prior to completing the experimental task.

### Physiological measures

Cardiovascular measures of sympathetic and parasympathetic cardiac control were derived from impedance cardiography (pre-ejection period (PEP)) and an electrocardiogram (high (respiratory) frequency (0.12–0.42 Hz) heart rate variability (HF HRV)). Data were scored minute by minute and then collapsed for each task.

PEP, derived from impedance cardiography, is the period between the electrical stimulation of the ventricular myocardium (Q wave of ECG) and the opening of the aortic valve. As PEP depends on the time development of intraventricular pressure, it is used as an index of cardiac contractility. Given variations in contractility are primarily under sympathetic control, PEP is used as a noninvasive measure of sympathetic influence of the heart [49,50]. Lower PEP values (in ms) represent higher levels of sympathetic activity. HF HRV is a rhythmic fluctuation of heart rate in the respiratory frequency band (respiratory sinus arrhythmia (RSA)) and has been demonstrated to be a relatively pure index of parasympathetic cardiac control [51].

The impedance cardiogram was collected using a four spot electrode configuration [52]. The electrocardiogram (ECG) was collected using the standard lead II configuration. The ECG and basal thoracic impedance (Z0) were measured using a Bionex system (MindWare Technologies LTD, Gahanna, OH). MindWare software was used to visually inspect all physiological data and to analyze the dZ/dt waveforms to obtain PEP from impedance. HR HRV was derived from ECG using spectral analysis of the interbeat interval series. The interbeat interval series was time sampled at 4 Hz (with interpolation) to yield an equal interval time series. This time series was detrended (second-order polynomial), end tapered, and submitted to a fast Fourier transformation. HF HRV spectral power was then integrated over the respiratory frequency band (0.12–0.42 HZ) and HF HRV is represented as the natural log of the heart period variance in the respiratory band (in ms^2^).

### Statistical analysis

To examine changes in the physiological measures and state anxiety and perceived stress over the course of the study by condition, repeated measures (time X condition) ANOVAs were run for each outcome measure. The sample size was determined to evidence a large effect size at 80% statiscal power.

To assess whether there were any differences between conditions in the amount of time individuals kept their foot in the water, a Cox proportional hazards regression model was run. For cold-pressor endurance time, foot withdrawal was defined as an event and individuals who endured the full 5 minutes were treated as censored in the analysis. We incorporated possible covariates into the models in a stepwise manner. To determine the power of the current data to detect an effect the size of any observed effects, we created simulated data sets from the current models and tested the proportion of simulated data sets the size of the observed effect.

## Results

### Sample composition

Of the 63 participants, 11 were excluded due to failure to completely submerge their foot in the water bath or confusion when taking their foot out resulting in inaccurate timing data. For the remaining 53 participants, 17 students were assigned to the breathing condition, 15 to the control, and 21 to the progressive muscle relaxation condition. Participants did not differ significantly on gender, age, income or ethnicity across conditions. Participants also did not differ significantly for depression, loneliness, trait and initial state anxiety, or initial stress across conditions.

### Questionnaire measures

**Fig 1:**
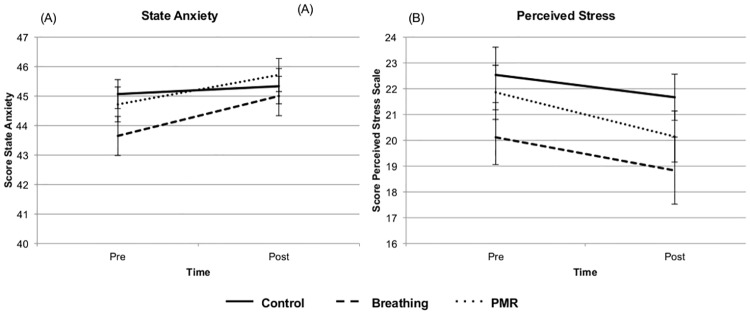
Effects of intervention on perceived anxiety and stress. (A) Perceived anxiety significantly increased over time, and (B) perceived stress significantly decreased, but there were no significant effects of intervention type on change in scores over time.

For state anxiety and perceived stress, 2 (pre/post manipulation) X 3 (condition) within subjects repeated measures ANOVAs were run to assess whether there were any changes in either measure after the manipulation. There was a significant main effect of time (pre/post) for state anxiety (F(1,51) = 7.91, p < 0.01), indicating a significant increase in participants’ anxiety after the task (Fig 1). There was no main effect of condition (F(2,51) = 0.88, p = 0.418) or interaction between condition and time (F(2,51) = 0.88, p = 0.420), suggesting condition had no influence on participants’ anxiety levels.

For perceived stress, there was also a significant main effect of Time (F(1,50) = 18.91, p < 0.001), with participants’ perceived stress decreasing after the manipulation (Fig 1). However, again there was no significant main effect of condition (F(2,50) = 1.46, p = 0.243) or significant interaction effect between condition and time (F(2,50) = 0.63, p = 0.537). Overall this suggests that while participants’ levels of perceived stress decreased over time, there were no differences in this change by condition, and participants’ anxiety levels did not change over the course of the experiment.

### Physiological measures

**Fig 2:**
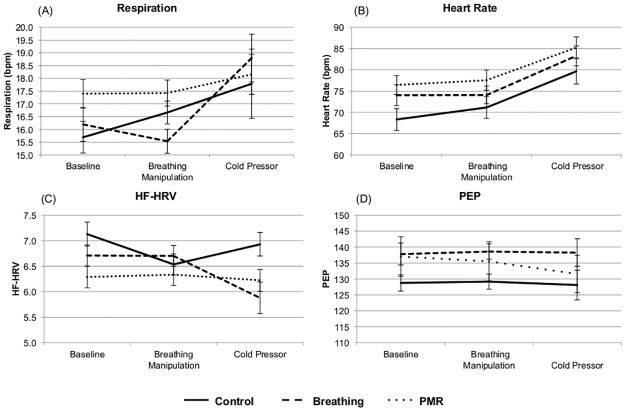
Effects of intervention on cardiac measures and respiration. Effects of intervention on cardiac measures and respiration: (A) Respiration, (B) Heart Rate, (C) HF-HRV, (D) PEP. There were no significant effects of intervention on any of the measures.

For mean heart rate, HF-HRV, respiration, and PEP, average values were calculated for the baseline period, condition period, and cold pressor. Using these values, 3 (baseline, condition, cold pressor) X 3 (condition) repeated measures ANOVAs were run to assess any changes in physiology over time by condition. For all measures but PEP, there was a significant effect of time on physiological change (HR: F(2,100) = 46.82, p < 0.001; HF-HRV: F(2,100) = 3.89, p < 0.05; Respiration: F(2,100) = 10.48, p < 0.001; PEP: F(2,96) = 0.75, p = 0.476; Fig 2). Post-hoc analyses suggested that these effects were driven by a significant increase between intervention (M = 74.64) and cold pressor (M = 83.03; Fisher-Hayter p < 0.001) for heart rate, a significant decrease between baseline (M = 6.66) and cold pressor (M = 6.31; Fisher-Hayter p < 0.01) for HF-HRV, and a significant increase between intervention (M = 16.52) and cold pressor (M = 18.26; Fisher-Hayter p < 0.001) for respiration. For all physiological measures, there was no main effect of condition (HR: F(2,50) = 2.28, p = 0.112; HF-HRV: F(2,50) = 2.39, p = 0.102); Respiration: F(2,50) = 0.79, p = 0.461; PEP: F(2,48) = 1.51, p = 0.231). For HF-HRV, however, there was a significant condition by time interaction (F(4,100) = 4.44, p < 0.01). Post hoc analysis indicated this was due to a significant decrease (Fisher-Hayter p < 0.05) in HF-HRV between intervention (M = 6.70) and cold pressor (M = 5.87) for the breathing group, while both for the control and PMR groups there were no significant post-hoc comparisons, indicating they remained stable across all tasks. There were no significant interactions for any of the other physiological measures (HR: F(4,100) = 0.38, p = 0.822; Respiration: F(4,100) = 2.11, p = 0.085; PEP: F(4,96) = 0.44, p = 0.777). Overall this suggests that the cold pressor induced significant increases in respiration and heart rate, and decreases in HF-HRV, as would be expected, but neither the breathing manipulation nor PMR produced significant differences in individuals’ physiological responses to the manipulation or cold pressor.

### Cold pressor

The initial Cox regression, incorporating just condition as a predictor of hazard produced no significant effects (Hazard Ratio = 0.991, p = 0.962), indicating that condition did not influence participants’ pain tolerance. To examine whether pain tolerance may be influenced by participants’ perceived pain, and whether perceived pain ratings interact with condition to produce differences in pain tolerance, we next incorporated pain ratings into the Cox regression model as a time-varying variable. This model did indicate a significant effect of perceived pain on pain tolerance (Hazard Ratio = 1.04, p < 0.01), suggesting participants with higher perceived pain are more likely to remove their foot from the water earlier, but this model did not change the effect of condition (Hazard Ratio = 1.52, p = 0.503), and there was no interaction effect (Hazard Ratio = 0.993, p = 0.507). The model incorporating both pain ratings and condition as predictors was a worse fit than modeling pain ratings alone.

As it was also hypothesized that changes in physiological measures due to the manipulation would influence participants’ pain tolerance, change scores between baseline and breathing conditions were calculated and incorporated into the model as time invariant variables. These produced no significant effects and did not change the effect of condition or improve overall model fit, suggesting that individual differences in physiological responsivity to the manipulation did not contribute to participants’ tolerance for pain (For all model specifications and results see S1 Appendix and S1 Table respectively).

While our simulated data sets demonstrated sufficient power for the observed null effect, this did not answer the question of whether we have sufficient power to observe a potential non-null effect if present. Given this concern, we also ran an ANOVA, for which we had sufficient power, to assess the effect of experimental condition on pain tolerance. Similarly to the survival analyses, we found no significant effects of condition on pain tolerance.

## Discussion

This study focused on illuminating the specific mechanisms through which subcomponents of mindfulness contribute to changes in pain tolerance and pain ratings. In contrast to previous research [1,20,21], we found no evidence for two relaxation subcomponents of mindfulness, in the form of deep breathing and PMR, altering individuals’ pain tolerance or self-reported pain. We also did not find any support that these types of interventions, at least within the context of a brief intervention, have physiological effects which contribute to individual differences in responses to the intervention. Overall, this suggests that a short term relaxation or focused breathing is not sufficient to influence participants’ experiences of pain.

There are several potential reasons why this study did not find expected differences in pain tolerance due to relaxation based manipulations. One explanation is that changing breathing and muscle relaxation simply do not influence experiences of pain. There is some evidence that deep breathing tasks that require attention (e.g. matching breaths to a visual stimulus, as employed in this task) do not significantly change pain thresholds, while those described as more relaxing, having individuals internally pace their breaths with audio instruction but no visual attention required, do significantly alter pain thresholds [20]. However, this does not explain the lack of difference across conditions in pain ratings, anxiety, and perceived stress changes, as the same study found both the attentive and relaxing intervention induced similar mood changes [20]. Nor does this explain the lack of effect for PMR, which rather than focusing on just changing breathing, focuses on changing muscle tension with brief reminders throughout the audio about to remember to breath but no specific instruction on how to alter breathing. Additionally, PMR has been demonstrated to be more reliably effective than other relaxation interventions in the context of chronic pain and injury pain [1], significantly influence nociceptive responses in novices [21], and modulate sympathetic responsivity [35]. Importantly, however, it is difficult to generalize and compare the findings in this study to those with chronic pain patients who experience long-term persistent pain in a naturalistic setting which has real threat value for these patients. This study employed a short term intervention in the context of an acute laboratory pain stimulus with no real threat value to healthy volunteers. Nonetheless, this still does not explain the lack of consistency in findings with studies that looked at nociceptive responses in novices [20,21].

A more likely explanation is that the intervention time period was too short to have an effect. While there have been a few studies which examined similarly short one-time interventions with novices and found some significant effects [25,53], the majority of studies addressing the question of whether relaxation interventions or mindfulness based training incorporating these aspects of relaxation influence experiences of pain have been conducted with long-term repeated intervention over the course of several weeks (most common 8 week intervention) [1,9]. It is likely that even in the case of the most basic type of relaxation, i.e., a focus on breathing, this initially requires effort and attention on the part of the individual, making it less relaxing, and after practice it becomes more automatic. Indeed, while there was a pattern towards decreased breathing rates in the breathing task condition, the fact there is not a significant condition by time interaction for respiration, suggests that the intervention was not as effective as expected.

It is also possible that relaxation tasks alone are not sufficient to induce changes in pain tolerance. Many of the mindfulness based interventions that have demonstrated effects on experimentally induced pain focus on changes in breathing in combination with teaching acceptance and awareness exercises [9,23]. These aspects may be key to modulating individuals’ experiences with pain. Indeed, it has been hypothesized that many of the effects of mindfulness/meditation interventions act through a cognitive restructuring, changing attention and self-regulatory processes [24,54]. However, more research is necessary to better understand the underlying neurobehavioral mechanisms through which these changes may be occurring [55]. Additionally, it is the case that these relaxation interventions alone, especially PMR, have previously demonstrated efficacy in altering perceptions of pain [1], making it more likely that the lack of effects are due to length of the intervention. Given the lack of consistency of activities across mindfulness interventions and their efficacy, it is important for future research to elucidate which aspects of these interventions are most important to achieving changes in not only pain, but also in other areas in which they are employed. Lastly, it is possible that the lack of effect was a result of a small sample size. However, our sample was comparable to that of previous studies that have found effects [20–23], and when analyses were re-run using a 3-way ANOVA comparing mean pain tolerance times, for which we had sufficient power (0.80) to detect a medium effect with our sample size, we still found no differences across conditions for participants pain tolerance. Despite this, future work should replicate and extend these findings with a larger sample size.

Overall this study provides no evidence for the hypothesis that changing breathing, common to most mindfulness interventions, is the key mechanism through which these interventions modulate individuals’ experiences of pain. Indeed we were unable to replicate any previous effects of either deep breathing or PMR on pain tolerance [20,21,25]. However, despite this, this study provides several important contributions to the understanding of relaxation interventions in the context of mindfulness. First, we have demonstrated that this brief of an intervention, 10 minutes, is not likely to have any large effects on novices’ experiences of pain, establishing a lower bound for efficacy of these types of interventions. Additionally, it suggests that the mechanisms involved in the beneficial effects of mindfulness interventions, are likely more complex than simply focusing on breathing or muscle tension thereby exerting physiological changes which then act to influence nociception and perceptions of pain. Given that these types of interventions are employed in treatment for a wide range of ailments, ranging from acute pain to depression to borderline personality disorder to supporting children experiencing early childhood trauma, it is important that more work focuses on what aspects of different mindfulness, meditation, and relaxation interventions, through direct comparison in a randomized control setting, actually contribute to observed effects on acute pain, as well as how long and how much practice is necessary to achieve these effects, in order to better understand the utility of different aspects of mindfulness as an intervention for experiences of pain, and more broadly their utility for treatment of a wide range of disorders.

## Supporting information

S1 Appendix Cox regression models. (DOCX)

S1 Data Data set used in manuscript. (XLSX)

S1 Table Cox regression model results: No significant effects of intervention, but pain ratings demonstrated a significant effect for people with higher pain ratings having faster time to removal of food from the water. Model A: Included only intervention as predictor; Model B: Included only pain ratings as predictor; Model C: Included intervention and pain ratings as predictors; Model D: Included intervention, pain ratings, change in PEP, RSA, HR and respiration from baseline to intervention, and baseline RSA as predictors. *p < 0.05, **p < 0.01, ***p < 0.001.
(DOCX)

## References

[1] Kwekkeboom K, Gretarsdottir E. Systematic review of relaxation intervention for pain. J Nurs Scholarsh. 2006;38: 269–277. 17044345 10.1111/j.1547-5069.2006.00113.x

[2] Manzoni GM, Pagnini F, Castelnuovo G, Molinari E. Relaxation training for anxiety: A ten-years systematic review with meta-analysis. BMC Psychiatry. 2008;8: 41 10.1186/1471-244X-8-41 18518981 PMC2427027

[3] Jain S, Shapiro SL, Swanick S, Roesch SC, Mills PJ, Bell I, et al A randomized controlled trial of mindfulness meditation versus relaxation training: effects on distress, positive states of mind, rumination, and distraction. Ann Behav Med. 2007;33: 11–21. 10.1207/s15324796abm3301_2 17291166

[4] Lancaster SL, Klein KP, Knightly W. Mindfulness and relaxation: A comparison of brief, laboratory-based interventions. Mindfulness (N Y). Mindfulness; 2016;7: 614–621.

[5] Baer RA. Mindfulness training as a clinical intervention: A conceptual and empirical review. Clin Psychol Sci Pract. 2003;10: 125–143.

[6] Wanden-Berghe RG, Sanz-Valero J, Wanden-Berghe C. The application of mindfulness to eating disorders treatment: A systematic review. Eat Disord J Treat Prev. 2011;19: 34–48. 10.1080/10640266.2011.533604 21181578

[7] Vøllestad J, Nielsen MB, Nielsen GH. Mindfulness- and acceptance-based interventions for anxiety disorders: A systematic review and meta-analysis. Br J Clin Psychol. 2012;51: 239–260. 10.1111/j.2044-8260.2011.02024.x 22803933

[8] Khoury B, Lecomte T, Fortin G, Masse M, Therien P, Bouchard V, et al Mindfulness-based therapy: A comprehensive meta-analysis. Clin Psychol Rev. 2013;33: 763–771. 10.1016/j.cpr.2013.05.005 23796855

[9] Chiesa A, Serretti A. Mindfulness-based interventions for chronic pain: A systematic review of the evidence. J Altern Complement Med. 2011;17: 83–93. 10.1089/acm.2009.0546 21265650

[10] Kabat-Zinn J. An outpatient program in behavioral medicine for chronic pain patients based on the practice of mindfulness meditation: Theoretical considerations and preliminary results. Gen Hosp Psychiatry. 1982;4: 33–47. 7042457 10.1016/0163-8343(82)90026-3

[11] Kakigi R, Nakata H, Inui K, Hiroe N, Nagata O, Honda M, et al Intracerebral pain processing in a Yoga Master who claims not to feel pain during meditation. Eur J Pain. 2005;9: 581–589. 10.1016/j.ejpain.2004.12.006 16139187

[12] Clark W, Clark S. Pain responses in Nepalese porters. Science (80-). 1980;209: 410–412. 10.1126/science.7384815 7384815

[13] Veehof MM, Trompetter HR, Bohlmeijer ET, Schreurs KMG. Acceptance- and mindfulness-based interventions for the treatment of chronic pain: A meta-analytic review. Cogn Behav Ther. 2016;45: 5–31. 10.1080/16506073.2015.1098724 26818413

[14] Reiner K, Tibi L, Lipsitz JD. Do mindfulness-based interventions reduce pain intensity ? A critical review of the literature. Pain Med. 2013;14: 230–242. 10.1111/pme.12006 23240921

[15] Zeidan F, Grant J, Brown C, Mchaffie J, Coghill R. Mindfulness meditation-related pain relief: Evidence for unique brain mechanisms in the regulation of pain. Neurosci Lett. Elsevier Ireland Ltd; 2012;520: 165–173. 10.1016/j.neulet.2012.03.082 PMC3580050 22487846

[16] Brown CA, Jones AK. Psychobiological correlates of improved mental health in patients with musculoskeletal pain after a mindfulness-based pain management program. Clin J Pain. 2013;29: 233–244. 10.1097/AJP.0b013e31824c5d9f 22874090

[17] Zeidan F, Gordon NS, Merchant J, Goolkasian P. The effects of brief mindfulness meditation training on experimentally induced pain. J Pain. Elsevier Ltd; 2010;11: 199–209. 10.1016/j.jpain.2009.07.015 19853530

[18] Brown CA, Jones AKP. Meditation experience predicts less negative appraisal of pain: Electrophysiological evidence for the involvement of anticipatory neural responses. Pain. 2010;150: 428–438. 10.1016/j.pain.2010.04.017 20494517

[19] Grant JA, Rainville P. Pain sensitivity and analgesic effects of mindful states in Zen meditators: A cross-sectional study. Psychosom Med. 2009;71: 106–14. 10.1097/PSY.0b013e31818f52ee 19073756

[20] Busch V, Magerl W, Kern U, Haas J, Hajak G, Eichhammer P. The effect of deep and slow breathing on pain perception, autonomic activity, and mood processing: An experimental study. Pain Med. 2012;13: 215–228. 10.1111/j.1526-4637.2011.01243.x 21939499

[21] Emery CF, France CR, Harris J, Norman G, VanArsdalen C. Effects of progressive muscle relaxation training on nociceptive flexion reflex threshold in healthy young adults: A randomized trial. Pain. 2008;138: 375–379. 10.1016/j.pain.2008.01.015 18291584

[22] Gard T, Hölzel BK, Sack AT, Hempel H, Lazar SW, Vaitl D, et al Pain attenuation through mindfulness is associated with decreased cognitive control and increased sensory processing in the brain. Cereb Cortex. 2012;22: 2692–2702. 10.1093/cercor/bhr352 22172578 PMC3968314

[23] Liu X, Wang S, Chang S, Chen W, Si M. Effect of brief mindfulness intervention on tolerance and distress of pain induced by cold-pressor task. Stress Heal. 2013;29: 199–204. 10.1002/smi.2446 22961992

[24] Gu J, Strauss C, Bond R, Cavanagh K. How do mindfulness-based cognitive therapy and mindfulness-based stress reduction improve mental health and wellbeing? A systematic review and meta-analysis of mediation studies. Clin Psychol Rev. Elsevier Ltd; 2015;37: 1–12. 10.1016/j.cpr.2015.01.006 25689576

[25] Arch JJ, Craske MG. Mechanisms of mindfulness: Emotion regulation following a focused breathing induction. Behav Res Ther. 2006;44: 1849–1858. 10.1016/j.brat.2005.12.007 16460668

[26] Cogan R, Kluthe KB. The role of learning in pain reduction associated with relaxation and patterned breathing. J Psychosom Res. 1981;25: 535–539. 7033520 10.1016/0022-3999(81)90107-0

[27] Dolbier CL, Rush TE. Efficacy of abbreviated progressive muscle relaxation in a high-stress college sample. Int J Stress Manag. 2012;19: 48–68.

[28] Hernandez-Reif M, Field T, Krasnegor J, Theakston H. Lower back pain is reduced and range of motion increased after massage therapy. Int J Neurosci. 2001;106: 131–145. 11264915 10.3109/00207450109149744

[29] Preyde M. Effectiveness of massage therapy for subacute low-back pain: A randomized controlled trial. CMAJ. 2000;162: 1815–1820. 10906914 PMC1231369

[30] Carson JW. Loving-kindness meditation for chronic low back pain: Results from a pilot trial. J Holist Nurs. 2005;23: 287–304. 10.1177/0898010105277651 16049118

[31] Travis F, Haaga DAF, Hagelin J, Tanner M, Nidich S, Gaylord-King C, et al Effects of Transcendental Meditation practice on brain functioning and stress reactivity in college students. Int J Psychophysiol. 2009;71: 170–176. 10.1016/j.ijpsycho.2008.09.007 18854202

[32] Critchley HD, Harrison NA. Visceral influences on brain and behavior. Neuron. 2013;77: 624–638. 10.1016/j.neuron.2013.02.008 23439117

[33] Gray MA, Minati L, Paoletti G, Critchley HD. Baroreceptor activation attenuates attentional effects on pain-evoked potentials. Pain. International Association for the Study of Pain; 2010;151: 853–861. 10.1016/j.pain.2010.09.028 PMC3038268 20965656

[34] Dworkin BR, Elbert T, Rau H, Birbaumer N, Pauli P, Droste C, et al Central effects of baroreceptor activation in humans : Attenuation of skeletal reflexes and pain perception. PNAS. 1994;91: 6329–6333. 8022781 10.1073/pnas.91.14.6329 PMC44195

[35] Hoffman JW, Benson H, Arns PA, Stainbrook GL, Young JB, Gill A. Reduced Sympathetic Nervous System Responsivity Associated with the Relaxation Response Published by: American Association for the Advancement of Science Stable URL: http://www.jstor.org/stable/1687600 Reduced Sympathetic Nervous System Responsivity Ass. 2009;215: 190–192. 10.1126/science.7031901 7031901

[36] Conrad A, Roth WT. Muscle relaxation therapy for anxiety disorders: It works but how? J Anxiety Disord. 2007;21: 243–264. 10.1016/j.janxdis.2006.08.001 16949248

[37] Spielberger CD, Gorsuch RL, Lushene R, Vagg PR, Jacobs GA. Manual for the State-Trait Anxiety Inventory. Palo Alto, CA: Consulting Psychologists Press; 1983.

[38] Cohen S, Kamarck T, Mermelstein R. A Global Measure of Perceived Stress [Internet]. Journal of Health and Social Behavior. 1983 pp. 385–396. 6668417

[39] Radloff LS. A self-report depression scale for research in the general population. Appl Psychol Meas. 1977;1: 385–401.

[40] Russell DW. UCLA Loneliness Scale (Version 3): Reliability, validity, and factor structure. Journal of Personality Assessment. 1996 pp. 20–40. 10.1207/s15327752jpa6601_2 8576833

[41] Porges SW. Body perception questionnaire Laboratory of Developmental Assessment, University of Maryland; 1993.

[42] McClernon FJ, Westman EC, Rose JE. The effects of controlled deep breathing on smoking withdrawal symptoms in dependent smokers. Addict Behav. 2004;29: 765–772. 10.1016/j.addbeh.2004.02.005 15135559

[43] Lucas SJE, Lewis NCS, Sikken ELG, Thomas KN, Ainslie PN. Slow breathing as a means to improve orthostatic tolerance: a randomized sham-controlled trial. J Appl Physiol. 2013;115: 202–11. 10.1152/japplphysiol.00128.2013 23681913

[44] Hapidou EG, De Catanzaro D. Sensitivity to cold pressor pain in dysmenorrheic and non-dysmenorrheic women as a function of menstrual cycle phase. Pain. 1988;34: 277–283. 3186275 10.1016/0304-3959(88)90123-6

[45] Johnson MH, Petrie SM. The effects of distraction on exercise and cold pressor tolerance for chronic low back pain sufferers. Pain. 1997;69: 43–48. 9060011 10.1016/s0304-3959(96)03272-1

[46] De Wied M, Verbaten MN. Affective pictures processing, attention, and pain tolerance. Pain. 2001;90: 163–172. 11166983 10.1016/s0304-3959(00)00400-0

[47] Stabell N, Stubhaug A, Flægstad T, Nielsen CS. Increased pain sensitivity among adults reporting irritable bowel syndrome symptoms in a large population-based study. Pain. 2013;154: 385–92. 10.1016/j.pain.2012.11.012 23320954

[48] Bisgaard T, Klarskov B, Rosenberg J, Kehlet H. Characteristics and prediction of early pain after laparoscopic cholecystectomy. Pain. 2001;90: 261–269. 11207398 10.1016/S0304-3959(00)00406-1

[49] Berntson GG, Cacioppo JT, Binkley PF, Uchino BN, Quigley KS, Fieldstone A. Autonomic cardiac control. III. Psychological stress and cardiac response in autonomic space as revealed by pharmacological blockades. Psychophysiology. 1994;31: 599–608. 7846220 10.1111/j.1469-8986.1994.tb02352.x

[50] Berntson GG, Cacioppo JT, Quigley KS. Autonomic determinism: The modes of autonomic control, the doctrine of autonomic space, and the laws of autonomic constraint. Psychol Rev. 1991;98: 459–487. 1660159 10.1037/0033-295x.98.4.459

[51] Berntson GG, Bigger JT JR, Eckberg DL, Kaufmann PG, Malik M, Nagaraja HN, et al Heart rate variability: origins, methods, and interpretive caveats. Psychophysiology. 1997;34: 623–48. Available: http://www.ncbi.nlm.nih.gov/pubmed/9401419 9401419 10.1111/j.1469-8986.1997.tb02140.x

[52] Sherwood A, Allen M, Fahrenberg J, Kelsey RM, Lovallow WR, van Doornen LJ. Methodological guidelines for impedance cardiography. Psychophysiology. 1990;27: 1–23. 2187214 10.1111/j.1469-8986.1990.tb02171.x

[53] Heenan A, Troje NF. Both physical exercise and progressive muscle relaxation reduce the facing-the-viewer bias in biological motion perception. PLoS One. 2014;9: 1–12. 10.1371/journal.pone.0099902 PMC4079562 24987956

[54] Shapiro SL, Carlson LE, Astin JA. Mechanisms of mindfulness. J Clin Psychol. 2006;62: 373–386. 10.1002/jclp.20237 16385481

[55] Tang Y-Y, Yang L, Leve LD, Harold GT. Improving executive function and its neurobiological mechanisms through a mindfulness-based intervention: Advances within the field of developmental neuroscience. Child Dev Perspect. 2012;6: 361–366. 10.1111/j.1750-8606.2012.00250.x 25419230 PMC4238887

