# Supplementary material for: Brief relaxation training is not sufficient to alter tolerance to experimental pain in novices
Source: PLoS One. 2017 May 11;12(5):e0177228. doi: 10.1371/journal.pone.0177228 (PMC5426711; doi:10.1371/journal.pone.0177228)
Supplement: S1 Appendix — (DOCX) [file pone.0177228.s001.docx]

**S1 Appendix: Cox regression models**

For all models, the hazard ratio represents the effect of one unit difference in associated predictor on raw hazard of foot removal from water during cold pressor.

**Model A:**

Includes only condition (breathing, PMR, control) as time-invariant predictor.

$$h\left( t_{ij} \right)=h_{o}(t_{j})e^{\beta_{1}{CONDITION}_{i}}$$

Where

$h\left( t_{ij} \right)$ = raw cumulative hazard of removal of foot

$h_{o}(t_{j})$ = baseline hazard function

$e^{\beta_{1}}$= hazard ratio for condition

**Model B:**

Includes only pain ratings as time-varying predictor.

$$h\left( t_{ij} \right)=h_{o}(t_{j})e^{\beta_{2}{RATING}_{ij}}$$

Where

$h\left( t_{ij} \right)$ = raw cumulative hazard of removal of foot

$h_{o}(t_{j})$ = baseline hazard function

$e^{\beta_{2}}$= hazard ratio for pain rating

**Model C:**

Includes both condition (time-invariant) and pain ratings (time-varying) as predictors

$$h\left( t_{ij} \right)=h_{o}(t_{j})e^{\beta_{1}{CONDITION}_{i}+ \beta_{2}{RATING}_{ij}+\beta_{3}{{CONDITION}_{i}\times RATING}_{ij}}$$

Where

$h\left( t_{ij} \right)$ = raw cumulative hazard of removal of foot

$h_{o}(t_{j})$ = baseline hazard function

$e^{\beta_{1}}$= hazard ratio for condition

$e^{\beta_{2}}$*=* hazard ratio for pain rating

$e^{\beta_{3}}$= hazard ratio for interaction of condition by pain rating

**Model D:**

Includes condition (time-invariant), pain ratings (time-varying), cardiac (HF-HRV, HR, and PEP) and respiration measures (time-invariant) as predictors.

$$h\left( t_{ij} \right)=h_{o}(t_{j})e^{\begin{aligned} \beta_{1}{CONDITION}_{i}+ \beta_{2}{RATING}_{ij}+\beta_{3}{{CONDITION}_{i}\times RATING}_{ij}+\beta_{4}{HR}_{i}+\beta_{5}{{CONDITION}_{i}\times HR}_{i}+\beta_{6}{{RATING}_{ij}\times HR}_{i}+\beta_{7}{{{CONDITION}_{i}\times RATING}_{ij}\times HR}_{i}+ \\ \beta_{8}{RESP}_{i}+\beta_{9}{{CONDITION}_{i}\times RESP}_{i}+\beta_{10}{{RATING}_{ij}\times RESP}_{i}+\beta_{11}{{{CONDITION}_{i}\times RATING}_{ij}\times RESP}_{i}+\beta_{12}{HFHRV}_{i}+\beta_{13}{{CONDITION}_{i}\times HFHRV}_{i}+ \\ \beta_{14}{{RATING}_{ij}\times HFHRV}_{i}+\beta_{15}{{{CONDITION}_{i}\times RATING}_{ij}\times HFHRV}_{i}+\beta_{16}{PEP}_{i}+\beta_{17}{{CONDITION}_{i}\times PEP}_{i}+\beta_{18}{{RATING}_{ij}\times PEP}_{i}+ \\ \beta_{19}{{{CONDITION}_{i}\times RATING}_{ij}\times PEP}_{i} \end{aligned}}$$

Where

$h\left( t_{ij} \right)$ = raw cumulative hazard of removal of foot

$h_{o}(t_{j})$ = baseline hazard function

$e^{\beta_{1}}$= hazard ratio for condition

$e^{\beta_{2}}$= hazard ratio for pain rating

$e^{\beta_{3}}$= hazard ratio for interaction of condition by pain ratings

$e^{\beta_{4}}$= hazard ratio for HR

$e^{\beta_{5}}$= hazard ratio for interaction of condition by HR

$e^{\beta_{6}}$= hazard ratio for interaction of pain ratings by HR

$e^{\beta_{7}}$= hazard ratio for interaction of condition by pain ratings by HR

$e^{\beta_{8}}$= hazard ratio for respiration

$e^{\beta_{9}}$= hazard ratio for interaction of condition by respiration

$e^{\beta_{10}}$= hazard ratio for interaction of pain ratings by respiration

$e^{\beta_{11}}$= hazard ratio for interaction of condition by pain ratings by respiration

$e^{\beta_{12}}$= hazard ratio for HF-HRV

$e^{\beta_{13}}$= hazard ratio for interaction of condition by HF-HRV

$e^{\beta_{14}}$= hazard ratio for interaction of pain ratings by HF-HRV

$e^{\beta_{15}}$= hazard ratio for interaction of condition by pain ratings by HF-HRV

$e^{\beta_{16}}$= hazard ratio for PEP

$e^{\beta_{17}}$= hazard ratio for interaction of condition by PEP

$e^{\beta_{18}}$= hazard ratio for interaction of pain ratings by PEP

$e^{\beta_{19}}$= hazard ratio for interaction of condition by pain ratings by PEP
