# Supplementary material for: Brief relaxation training is not sufficient to alter tolerance to experimental pain in novices
Source: PLoS One. 2017 May 11;12(5):e0177228. doi: 10.1371/journal.pone.0177228 (PMC5426711; doi:10.1371/journal.pone.0177228)
Supplement: S1 Table — Model A: Included only intervention as predictor; Model B: Included only pain ratings as predictor; Model C: Included intervention and pain ratings as predictors; Model D: Included intervention, pain ratings, change in PEP, RSA, HR and respiration from baseline to intervention, and baseline RSA as predictors. *p < 0.05, **p < 0.01, ***p < 0.001. (DOCX) [file pone.0177228.s003.docx]

| **S1 Table: Cox regression model results** | | | |
| --- | --- | --- | --- |
| **Model** | **Predictor** | **Hazard Ratio** | **Asymptotic Standard Error** |
| Model A | Intervention | 0.991 | 0.187 |
| Model B | Pain Rating | 1.032*** | 0.007 |
| Model C | Intervention | 1.522 | 0.627 |
|  | Pain Rating | 1.04** | 0.014 |
|  | Intervention*Pain Rating | 0.994 | 0.010 |
| Model D | Intervention | 3.715 | 0.951 |
|  | Pain Ratings | 1.080** | 0.025 |
|  | Change PEP | 0.631 | 0.244 |
|  | Change RSA | 0.868 | 2.303 |
|  | Change HR | 0.621 | 0.480 |
|  | Change Respiration | 1.406 | 0.662 |
|  | Intervention*Pain Ratings | 0.977 | 0.015 |
|  | Intervention*PEP Change | 1.322 | 0.212 |
|  | Pain Ratings*PEP Change | 1.008 | 0.004 |
|  | Intervention*RSA Change | 2.010 | 1.903 |
|  | Pain Ratings*RSA Change | 1.024 | 0.033 |
|  | Intervention*HR Change | 1.191 | 0.356 |
|  | Pain Ratings*HR Change | 1.007 | 0.007 |
|  | Intervention*Respiration Change | 0.647 | 0.499 |
|  | Pain Ratings*Respiration Change | 0.996 | 0.009 |
|  | Intervention*Pain Ratings*PEP Change | 0.996 | 0.003 |
|  | Intervention*Pain Ratings*RSA Change | 0.971 | 0.029 |
|  | Intervention*Pain Ratings*HR Change | 0.997 | 0.005 |
|  | Intervention*Pain Ratings*Respiration Change | 1.006 | 0.007 |

**S1 Table:** No significant effects of intervention, but pain ratings demonstrated a significant effect for people with higher pain ratings having faster time to removal of food from the water. Model A: Included only intervention as predictor; Model B: Included only pain ratings as predictor; Model C: Included intervention and pain ratings as predictors; Model D: Included intervention, pain ratings, change in PEP, RSA, HR and respiration from baseline to intervention, and baseline RSA as predictors. *p < 0.05, **p < 0.01, ***p < 0.001
